# Mental health literacy and help-seeking intention among Chinese elite athletes: the mediating roles of stigma and social support

**DOI:** 10.3389/fpsyg.2024.1332343

**Published:** 2024-09-12

**Authors:** Danran Bu, Chun-Qing Zhang, Wei Liang, Zhe Han, Nian Yi, Ning Su, Zhijian Huang

**Affiliations:** ^1^School of Physical Education, Hubei University, Wuhan, China; ^2^Department of Psychology, Sun Yat-sen University, Guang Dong, China; ^3^School of Physical Education, Shenzhen University, Shenzhen, China; ^4^Key Laboratory of General Administration of Sport of China, Hubei Institute of Sport Science, Wuhan, China

**Keywords:** mental health literacy, help-seeking intention, stigma, social support, Chinese elite athletes

## Abstract

**Objectives:**

Considering the importance of mental health help-seeking, researchers have closely examined the relationship between mental health literacy (MHL) and help-seeking intention (HSI). Furthermore, the high impact of stigma and the potential value of social support on HSI have been recognised. However, the relationship between these variables has not been fully tested within the context of Chinese elite athletes. This study addressed this gap by examining the relationship between MHL and HSI. Furthermore, it explored the mediating effects of public stigma, self-stigma, and social support on the relationship between MHL and HSI among Chinese elite athletes.

**Methods:**

450 Chinese elite athletes (*M*_age_ = 18.12, *SD* = 3.20, 46.2% female) self-reported their MHL, public stigma, self-stigma, social support, and HSI. Mediation analysis was conducted using the bootstrap approach of the PROCESS macro version 3.3 of SPSS 25.

**Results:**

The results showed a significant positive correlation between MHL and HSI (*r* = 0.348). The results also demonstrated that MHL was a predictor of HSI (*β* = 0.337, 95%*CI* [0.249, 0.425], *p* < 0.001). Furthermore, 1) the indirect effect of MHL → public stigma→HSI was 0.024 and a 95%*CI* [0.003, 0.053]. Specifically, MHL predicted public stigma (Estimate = −0.151, 95%*CI* [−0.187, −0.045], *p* < 0.001), and public stigma predicted HSI (Estimate = −0.161, 95%*CI* [− 0.549, −0.164], *p* < 0.001); 2) the indirect effect of MHL → self-stigma→ HSI was 0.016 and 95%*CI* [0.002, 0.038]. Specifically, MHL predicted self-stigma (Estimate = −0.137, 95%*CI* [−0.069, −0.013], *p* < 0.01), and self-stigma predicted HSI (Estimate = −0.120, 95%*CI* [−1.181, −0.186], *p* < 0.01); as well as 3) the indirect effect of MHL → social support→HSI was 0.029 and a 95%*CI* [0.009, 0.055]. Specifically, MHL predicted social support (Estimate = 0.208, 95%*CI* [0.018, 0.047], *p* < 0.001), and social support predicted HSI (Estimate = 0.141, 95%*CI* [0.578, 2.442], *p* < 0.01). Additionally, the direct effects from MHL to HSI is (β = 0.452, 95%*CI* [0.304, 0.600], *p* < 0.001).

**Conclusion:**

Our findings provide empirical support for the roles of public stigma, self-stigma, and social support as mechanisms of behavioural change in MHL interventions. These factors increase HSI among elite athletes. Future studies should further test these mediating effects using experimental designs.

## Introduction

Elite athletes experience mental health issues during their competitive careers or when they transition to their post-athletic careers (Allen and Hopkins, 2015; Gouttebarge et al., 2016; Schinke et al., 2018; Stambulova and Ryba, 2014). Research has shown that elite athletes struggle with mental health issues, which can have a detrimental impact on their performance and well-being, as well as cause significant personal, social, and financial burdens (Dowell et al., 2020; Schinke et al., 2018). Several organisations have thus highlighted the importance of mental health among elite athletes (Gouttebarge et al., 2021; Henriksen et al., 2019; Reardon et al., 2019; Si et al., 2021).

Elite athletes suffer from serious mental health issues. For example, research has found that common mental health issues include depression, anxiety, and sleeping problems (Chang et al., 2020; Reardon et al., 2019). However, elite athletes often avoid or delay obtaining professional help (Gulliver et al., 2012a). Help-seeking in the context of mental health refers to a process of adaptive coping in which individuals look for external help to address a mental health concern (Rickwood and Thomas, 2012). In China, a study by (Guo, 2008), found that 86% of elite athletes had never received professional mental health assistance. The elite sports environment can expose athletes to barriers that affect their attitudes, reducing their willingness to seek help (Breslin et al., 2022; Bu et al., 2020). Help-seeking has thus emerged as a high priority for research, policy, and programme initiatives (Rickwood and Thomas, 2012).

A systematic review found that the main barriers to help-seeking in elite sports were poor mental health literacy (MHL) and the high level of stigma associated with mental health (Gulliver et al., 2010). MHL refers to understandings and perspectives of mental disorders, which facilitate their identification, treatment, or prevention (Jorm et al., 1997). As the level of MHL in the general population worldwide is low, improving MHL is essential to normalise, prevent, and detect mental health issues (Jorm, 2000; Moesch et al., 2018). A similar situation exists among elite athletes, and better action for improvement is required (Breslin et al., 2018). For instance, a qualitative research conducted by Gulliver et al. (2012a) found that elite athletes were unaware of the signs of mental disorders, such as the difference between fatigue caused by physical exhaustion from training and depression or anxiety. It has been demonstrated that 70% of Chinese elite athletes have a low level of MHL, which means that many athletes do not know how to recognise mental health issues (Guo, 2008). Another study conducted by Han et al. (2019) found that 92% of Chinese elite athletes had a low to medium level of MHL, requiring improvement. Furthermore, previous studies have demonstrated that a lower level of MHL was associated with a higher level stigma towards mental health issues and a lower degree of social support when individuals might ask for assistance (Jung et al., 2017; Svensson and Hansson, 2016). For instance, a study has revealed a significant positive correlation between MHL and help-seeking intention (HSI) in Korea college students (*r* = 0.27) (Kim et al., 2020). Another study (Yang et al., 2023) has demonstrated that a significant negative correlation between MHL and stigma in Chinese adults (*r* = −0.102). Yang et al. (2023) further explored the indirect effect from MHL → stigma →help-seeking attitudes was (*β* = 0.012, 95%*CI* [0.019, 0.125]). Additionally, bivariate correlation analysis has demonstrated that MHL was positively associated with social support in American employees (*r* = 0.19) (Jung et al., 2017). In summary, the relationships among MHL, stigma, social support and HSI have been documented in different population.

Additionally, the higher level of stigma attached to mental health problems has been noted as one of the primary barriers to obtaining help in elite athletes (Abram et al., 2008; Larkin et al., 2017). Stigma refers to the negative attitudes, sentiments, and actions directed at people or groups who exhibit traits or act in ways that society deems unacceptable and/or insufficient (Wahto et al., 2016; Watson, 2006). Public stigma and self-stigma are the two types of stigmas. Public stigma is the public perception that the stigmatised group is socially unacceptable (Corrigan, 2004). The main form of public stigma experienced by elite athletes is that their coaches, teammates, family members, and even their competitors may think they are weak (Bauman, 2016; Gulliver et al., 2010). For instance, when coaches hold a negative view of mental health issues, this can affect how athletes participate and perform in sports, as well as their general well-being (Mazzer and Rickwood, 2015). Furthermore, researchers found that male athletes frequently experienced peer devaluation when they sought assistance from sports psychologists (Linder et al., 1989). Self-stigma is the devaluation of a person’s self-esteem and self-efficacy that occurs when they experience mental health issues resulting from public stigma (Corrigan, 2004). To avoid being perceived as incompetent or having a negative self-image, elite athletes may be reluctant to report their mental health issues, which may lead to a reluctance to ask for assistance (Lannin et al., 2016). Higher degrees of public-and self-stigmas may impede decision-making and the process of choosing services (Cauce et al., 2002). A qualitative study has revealed that the athletes expressed the opinion that the general public would probably perceive athletes who seek treatment for mental health problems as weak and unworthy. Additionally, they also stated that they would feel guilty and shame if they experienced mental health issues or if others found out that they had sought treatment for mental health issues (Bu et al., 2024). Thus, both public stigma and self-stigma can impact the HSI of athletes.

Social support refers to the individuals within a support network providing various types of assistance to the one attempting to manage or negate a stressful situation (Heerde and Hemphill, 2018). These kinds of social ties and supportive relationships with others are essential to mental health (Turner and Brown, 2010). It can facilitate help-seeking and should be complemented by various support resources. For athletes, support generally comes from coaches, family members, teammates, and psychologists (Coyle et al., 2017). Most athletes who have positive attitudes towards mental health issues believe that they can benefit from those around them. This kind of support can lead to adaptive outcomes, including having conversations, creating a loving environment, and offering useful advice (Dorsch et al., 2017; Hurley et al., 2017). Additionally, athletes’ mental health might improve if they have long-time established and dependable relationships with supportive individuals (Bu et al., 2023; Sebbens et al., 2016). The physical and psychological safety environment in sports can also help athletes pay attention to their personal development (Fan et al., 2022). Therefore, strong and positive social support can promote discussion of mental health and related issues, as well as help-seeking (Rickwood et al., 2005; Swann et al., 2018).

A potential approach to overcoming obstacles to seeking assistance is raising the MHL of individuals in elite environment, such as athletes, coaches, and support staff (Bu et al., 2023; Sebbens et al., 2016). Specifically, Gulliver et al. (2012b) have revealed that the Internet-based MHL intervention could enhance young elite athletes MHL level, help-seeking attitudes and intentions, as well as reduce stigmas. Additionally Bu et al. (2023), have conducted an MHL intervention focused on team officials and staff in Chinese elite sports. The results have demonstrated the intervention could raise their MHL levels, lower the stigma associated with mental health issues, and improve help-seeking attitudes. This could also provide a supportive environment for those who might have mental health issues in elite athletes. In summary, athletes and supporting staff with low MHL may have detrimental effects on athletes’ mental health, such as difficulty recognizing and diagnosing symptoms, postponing seeking assistance or not seeking treatment, and increased stigma (Hurley et al., 2018; Jorm, 2000). Additionally, the enhancements of MHL could assist athletes in providing social support, such as offering useful advice when suffering from mental health issues (Hurley et al., 2017). These kinds of results could provide empirical connections support among above variables.

The distinct nature of the culture and sports governing model in China contrasts sharply with Western approaches (Bu et al., 2023). Firstly, because of their traditional culture, Chinese individuals frequently favor independence or turn to traditional Chinese medicine for mental health care, with a belief in the punishment for the wrongdoing of ancestors (Wei et al., 2020; Wong et al., 2004). This not only leads to a lower level of MHL in individuals, but may also lead to the recurrence and worsening of mental health problems (Wei et al., 2020; Wong et al., 2004). Secondly, in contrast to Western nations with more individualistic cultures, China’s more collectivistic society may exhibit higher levels of both public and self-stigmas linked to mental health concerns (Furnham and Chan, 2004; Griffiths et al., 2006). Furthermore, compared to Western nations, the sports systems in China are very dissimilar. In particular, the sports training centres supervise and receive all funding from the Chinese government for the sports system (Si and Jiang, 2011). Nonetheless, the majority of athletes in Western nations receive their training in publicly managed training facilities and sports clubs, with some government funding (Si et al., 2021). This might lead to all entourage in the Chinese sports environment focusing only on performance. This environment might potential impact mental health and personal growth of athletes (Henriksen et al., 2019). Thirdly, the insufficient knowledge of MHL of the entourage of athletes, such as team officials, support staff, parents, and coaches (Bu et al., 2023), the social support might not be enough from those individuals. All of these factors might further impact mental health help-seeking. In conclusion, there are notable differences between China and Western countries with regard to the mental health and mental health framework.

Owing to the significance of seeking mental health assistance, the relationship between MHL and HSI has received considerable attention. The avoidance of help-seeking when suffering from mental health issues can be explained by the psychological theory of behaviour change (Breslin et al., 2016). The help-seeking theory provides a solid theoretical foundation and guide for help-seeking interventions in mental health (Rickwood et al., 2005). It conceptualises help-seeking as a four-step framework process model that integrates individual and psychological factors that facilitate or hinder the help-seeking process at the micro level. Help-seeking is described as a process of positive search and the use of social relationships to deal with personal problems through a formal or informal approach. The four steps of help-seeking theory are (1) awareness and appraisal of problems, (2) expression of symptoms and need for support, (3) availability of sources of help, and (4) willingness to seek out and disclose to sources (Rickwood et al., 2005). According to this theory, the first step is an important aspect of MHL affecting early detection and predicting help-seeking behaviour (Wright et al., 2007). The second step is associated with stigmas. If athletes are reluctant to express mental health problems due to self-stigma or public stigma, this can affect their willingness to seek help. The availability of sources of help might involve informal and formal sources, which are associated with social support. Therefore, there is a theoretical connection among MHL, stigmas, social support, and HSI.

Additionally, previous research has suggested that MHL is positively correlated with HSI (O’Connor and Casey, 2015; Wilson et al., 2005; Smith and Shochet, 2011). However, despite promising interventions based on the correlation between MHL and HSI, the relationship is not fully understood (Hansson and Markström, 2014; Pinto-Foltz et al., 2011; White and Casey, 2016). Specifically, it is unclear how some vital components, including public stigma, self-stigma, and social support, affect the relationship between MHL and help-seeking among Chinese elite athletes. Only one study focused on the relationship between these variables, which has examined the relationship between MHL, stigma, and HSI in the United States (Rafal et al., 2018). However, this study only recruited male college athletes as participants, which may limit its application to female athletes and elite athletes. Furthermore, this study did not fully test the relationship between MHL, stigma, and HSI, but only examined how they differed between undergraduate and graduate students, and between race and major classification.

**Figure 1:**
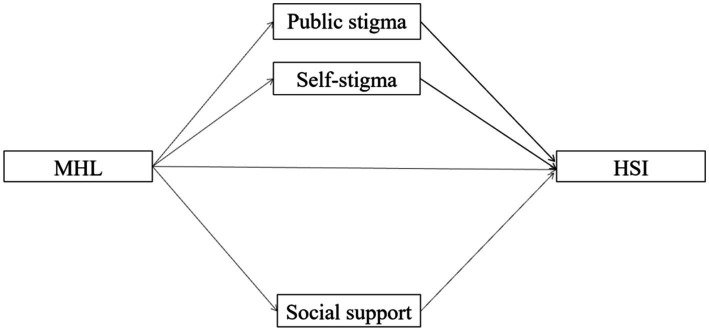
Hypothesis Model of MHL, Public- and Self-Stigmas, Social Support and HSI. MHL, mental health literacy, HSI, help-seeking intention.

The low level of MHL and the high levels of public stigma and self-stigma among Chinese elite athletes have a potentially negative impact on seeking assistance. It is thus crucial to explore the relationship between MHL and HSI in Chinese elite athletes to promote mental health help-seeking. The objective of this study is to examine the relationship between MHL and HSI, as well as the mediating roles of public stigma, self-stigma, and social support in the relationship between MHL and HSI among Chinese elite athletes (see Figure 1). Chinese elite athletes are those who have a province-level elite athlete certificate (Wu et al., 2013). We tested four hypotheses. Given that MHL is the main factor that affects help-seeking in elite sports, Hypothesis 1 posits a significant positive correlation between MHL and HSI. Considering that higher levels of public stigma and self-stigma may negatively impact athletes’ mental health help-seeking, Hypothesis 2 posits a negative and significant indirect effect of public stigma on the relationship between MHL and athletes’ HSI. Hypothesis 3 posits a negative and significant indirect effect of self-stigma on the relationship between MHL and athletes’ HSI. As discussed, strong social support may increase the likelihood that athletes will seek help. Therefore, Hypothesis 4 posits a positive and significant indirect effect of social support on the relationship between MHL and athletes’ HSI.

## Methods

### Participants

The participants were Chinese elite athletes recruited via convenience sampling from different sports training centres in two Chinese provinces. The following were the inclusion criteria for the participants: (1) elite athletes who have competed above provincial-level, such as the provincial, national, or international level; (2) active athletes able to attend daily training and participate in competitions. The exclusion criteria was elite athletes with disability. Based on previous studies, MedPower (Kenny, 2017) was used to calculate to detect a moderate indirect effect (ab; regression coefficients for a and b paths = 0.25, a power of 0.8, and an alpha of 0.05). The required sample size was 163. To boost power, we opted to oversample in this study. A total of 450 athletes participated in the study.

The participants ranged in age from 16 to 29 years (*M* = 18.12, *SD* = 3.20), with 242 men and 208 women. Their training experience ranged from 2 years to 19 years (*M* = 7.90, *SD* = 3.44). Their average training days per week were 5.89 days (*SD* = 0.50), with an average daily training time of 5.84 h (*SD* = 1.33). The athletes came from 14 sports – shooting, archery, athletics, badminton, table tennis, basketball, football, volleyball, swimming, synchronized swimming, modern pentathlon, gymnastics, free wrestling, and weightlifting. Four hundred fifty athletes participated in individual events and 32 in team events. There were 163 provincial-level athletes, 123 national-level athletes, and 164 international-level athletes.

### Procedure

This study used a cross-sectional approach to obtain data from athletes. Before the start of the study, the study was approved by the Research Ethics Committee of the Research Ethics Committee of Hubei institute of Sport Science. The researchers then contacted the officials of nine training centres in two provinces and invited athletes from different events to participate in the study. All athletes signed informed consent forms. For athletes under 18 years of age, their parents also signed informed consent forms.

The data were collected from May to August 2021. Before completing the Chinese version of the questionnaire, a pilot test with all the above questionnaires was conducted with elite athletes to verify the readability and clarity of the items. During the answering process, an experienced sports psychologist explained the items to the participants to guarantee the readability of the questionnaires. The participants answered the questions using the *Questionnaire Star* website and their smartphones. If there is a question that has not been answered, there will be a hint on the interface. Therefore, there is no missing data in our study. During the process, if a participant was unsure of the meaning of an item, the sports psychologist explained it further. Answering the questionnaire took about 10 ~ 16 min. To control the quality of the data, all data with a response time of less than 200 s were deleted (Jaeger and Cardello, 2022). Four hundred sixty-four athletes were invited, and 450 of them have answered. The response rate was 97.98%.

### Measures

MHL was measured using the Chinese version of the Mental Health Literacy Scale (MHLS) (Han et al., 2019). The Chinese version of the 33-item MHLS has previously been tested in the elite athlete population (aged 12 to 31). It has a minimum score of 33 and a maximum score of 150, with a higher score indicating a higher level of MHL. Questions on a 4-point scale are rated (1 = unhelpful/very unlikely to 4 = helpful/very likely), and those on a 5-point scale are rated (1 = definitely unwilling/strongly disagree to 5 = definitely willing/strongly agree) (e.g., ‘I am sure where to acquire information about mental illness.’). The internal consistency of this scale is 0.704 and the test–retest reliability is 0.763, with good construct validity. In this study, the internal consistency of the scale was Cronbach’s α = 0.769.

HSI was assessed with the Intentions to Seek Counseling Inventory (Cash et al., 1975; Hao and Liang, 2011), which contains 16 items and three dimensions: mental health and interpersonal relationship issues, performance issues, and substance abuse issues. A 6-point Likert scale (1 = very unlikely to 6 = very likely) was applied to evaluate HSI (e.g., ‘I will seek professional help if I experience sleep difficulties’). The Chinese version was tested on university students and elite athletes (aged 12 to 31), with internal consistency reliability ranging from 0.71 to 0.92 (Han et al., 2019; Hao and Liang, 2011). In this study, the internal consistency of this scale was Cronbach’s *α* = 0.850.

Stigma associated with mental health issues was assessed using the Perceived Devaluation–Discrimination Scale (Gao and Li, 2013; Link, 1987), which contains 15 items and assesses two different forms of stigma: public stigma and self-stigma. Each question was rated by the participants using a 5-point Likert scale (1 = totally disagree, 5 = totally agree, e.g., ‘People think that seeking psychiatric services is a mark of personal failure’), with a higher score indicating more stigma associated with mental health issues. The Chinese version was examined in the general population and on elite athletes (aged 12 to 31). The internal consistency reliability of the scale ranges from 0.71 to 0.81 (Gao and Li, 2013; Han et al., 2019). In this study, Cronbach’s α was 0.743, indicating acceptable internal consistency.

Given the importance of families, coaches, friends, teammates, and psychologists involved in mental health support, social support was also evaluated. The measure of social support was adapted from previous research to assess the concerns of significant others in relation to elite athletes seeking support from mental health professionals (Han et al., 2019; Rafal et al., 2018). Social support was assessed with one item: ‘If you had a mental health issue, would your parents/coaches/friends/teammates/psychologists encourage you to see a mental health professional?’ The measure was rated on a 5-point Likert-type scale, ranging from 1 “not at all,” 2 “somewhat,” 3 “moderate,” 4 “often,” to 5 “very,” with a higher score indicating a higher level of social support.

### Data analysis

The demographic variables of the participants and the internal consistency coefficient of each questionnaire were statistically calculated using IBM SPSS 25 (Armonk, NY; IBM Corp 2017). Three dependent variables – athlete public stigma, self-stigma, and social support – were tested through three mediation models. MHL was the predicting variable, while public stigma, self-stigma, and social support were the mediators.

A mediation analysis was then performed using the PROCESS macro version 3.3 of SPSS 25 developed by Hayes (2013). Tests of mediation roles were conducted using the bootstrap approach with 5,000 resamples recommended by Preacher and Hayes (2008) and Streukens and Leroi-Werelds (2016) and a 95% confidence interval (CI) (Baron and Kenny, 1986). 5,000 replications have been used throughout this study as more bootstrapped samples to enhance estimation (Banjanovic and Osborne, 2016). All variables were standardized, and gender, age, sport type, training years, training level, training days per week, and hours were controlled for in the mediation models. Specifically, considering gender, males are less likely to seek help than females, because the requirements of traditional masculine norms might exist in the male population (Levant et al., 2013; Seidler et al., 2016). With regard to age, previous research has demonstrated that young people are less likely to seek help than adults (Rickwood et al., 2007). Furthermore, considering sport types, as athletes in individual sports have been shown to experience a higher incidence of mental health symptoms than athletes in team sports (Pluhar et al., 2019; Seidler et al., 2016; Yang, 2015), it might impact help-seeking assistance. Additionally, training years, training level, and days of training per week are highly associated with stress (Bu, 2021). As stress is the main risk factor for mental health in elite athletes, it might impact the help-seeking, which is the main coping resource when they suffer from mental health issues (Bu, 2021). Therefore, the above variables were controlled for HSI.

The indirect effect was considered significant if zero was not involved in the 95% *CI* of its effect size. Standardized estimates for total and specific indirect effects of mediators were calculate. *R_med_^2^* was used to calculate the effect size (Fairchild et al., 2009).

## Results

The descriptive statistics and correlations between MHL, public stigma, self-stigma, stigma, social support, and HSI are shown in Table 1. The results showed a significant negative correlation between MHL and public stigma (*r* = −0.126), self-stigma (*r* = −0.141), and stigma (*r* = −0.159), and a significant positive correlation between MHL and social support (*r* = 0.202) and HSI (*r* = 0.348). Public stigma was significantly positively linked with self-stigma (*r* = 0.188), and stigma (*r* = 0.942), and negatively linked with social support (*r* = −0.108) and HSI (*r* = −0.230). Self-stigma was significantly positively linked with stigma (*r* = 0.506), and significantly negatively linked with social support (*r* = −0.187) and HSI (*r* = −0.222). Stigma was negatively linked with social support (*r* = −0.391) and HSI (*r* = −0.410). Social support was significantly positively linked with HSI (*r* = 0.248). Based on the Shapiro–Wilk test, the results of MHL, public stigma, self-stigma, social support, and HSI did not conform to the normal distribution (*p* < 0.05), nonparametric tests were applied.

**Table 1:** Means, standard deviations, skewness, kurtosis, internal consistency coefficients, and correlations of the study variables (*N* = 450).

Variable	M	SD	Ske	Kur	α	1	2	3	4	5	6
1 MHL	87.98	7.28	−0.470	1.330	0.769	1					
2 Public Stigma	30.22	5.58	−0.802	0.850	0.747	−0.126**	1				
3 Self-Stigma	7.03	2.17	−0.066	−0.310	0.679	−0.141**	0.188***	1			
4 Stigma	37.26	6.35	−0.737	0.843	0.743	−0.159**	0.942***	0.506***	1		
5 Social Support	2.49	1.15	0.341	−0.159	-	0.202***	−0.108*	−0.187***	−0.391**	1	
6 HSI	54.23	12.34	−0.111	−0.827	0.850	0.348***	−0.230***	−0.222***	−0.410**	0.248***	1

M, Mean; SD, Standard Deviations; Ske, Skewness; Kur, Kurtosis; α, Cronbach’s alpha coefficient; MHL, Mental health literacy; HSI, Help-seeking intention; ^*^ *p* < 0.05, ^**^ *p* < 0.01, ^***^ *p* < 0.001.

Harman’s single-factor test was applied to check for common method bias (Zhou and Long, 2004). The results showed that 19 factors had feature values greater than 1, which explained 59.40% of the total variance. Among the 19 factors, 12.23% of the variance was explained by the largest factor, or 40% below the critical value. Therefore, it may be said that this study did not have a common method bias problem.

Multicollinearity tests were performed with HSI as the dependent variable and MHL, public stigma, self-stigma, and social support as independent variables. The resulting regression equation was statistically significant (*F* = 27.579, *p* < 0.001). The four variables explained 19.9% of the variance in HSI, and the adjusted amount explained 19.1% of the variance in HSI. The results of the multicollinearity analysis showed that there was no severe multicollinearity between the variables (tolerance = 0.929–0.951, variance inflation factor = 1.051–1.077, eigenvalues = 0.003–4.748, conditional indicator = 1.000–40.887). Therefore, mediation analysis could be performed to further explore the relationships between these variables.

The indirect effect test procedure was then used to test the mediating roles of public stigma, self-stigma, and social support in the relationship between MHL and HSI. The total, direct, and indirect effects of public stigma, self-stigma, and social support on the relationship between MHL and HSI in elite athletes are shown in Table 2. Regression analyses were conducted with MHL as the independent variable and HSI as the dependent variable. The results showed that MHL was a predictor of HSI (*β* = 0.337, 95%*CI* [0.249, 0.425], *p* < 0.001), explaining 14.1% of the variance in HSI when gender, age, sport type, training years, training level, training days per week, and hours were controlled for.

**Table 2:** Effects of MHL on HSI via the mediators of public stigma, self-stigma, and social support among Chinese elite athletes (*N* = 450).

Effects	ES	BootSE	95%*CI*	
			LL	UL
Total effects				
MHL → HSI	0.337	0.045	0.249	0.425
Direct effects				
MHL → HSI	0.267	0.045	0.179	0.354
Indirect effects				
MHL → Public stigma → HSI	0.024	0.013	0.003	0.053
MHL → Self-stigma → HSI	0.016	0.009	0.002	0.038
MHL → Social support → HSI	0.029	0.016	0.009	0.055

MHL, mental health literacy; HSI, help-seeing intention; ES, effect size; SE, standard error; CI, confidence interval; LL, lower limit; UL, upper limit; Gender, age, sports type, training years, training level, days of training per week and hours of were controlled in the mediation models.

**Figure 2:**
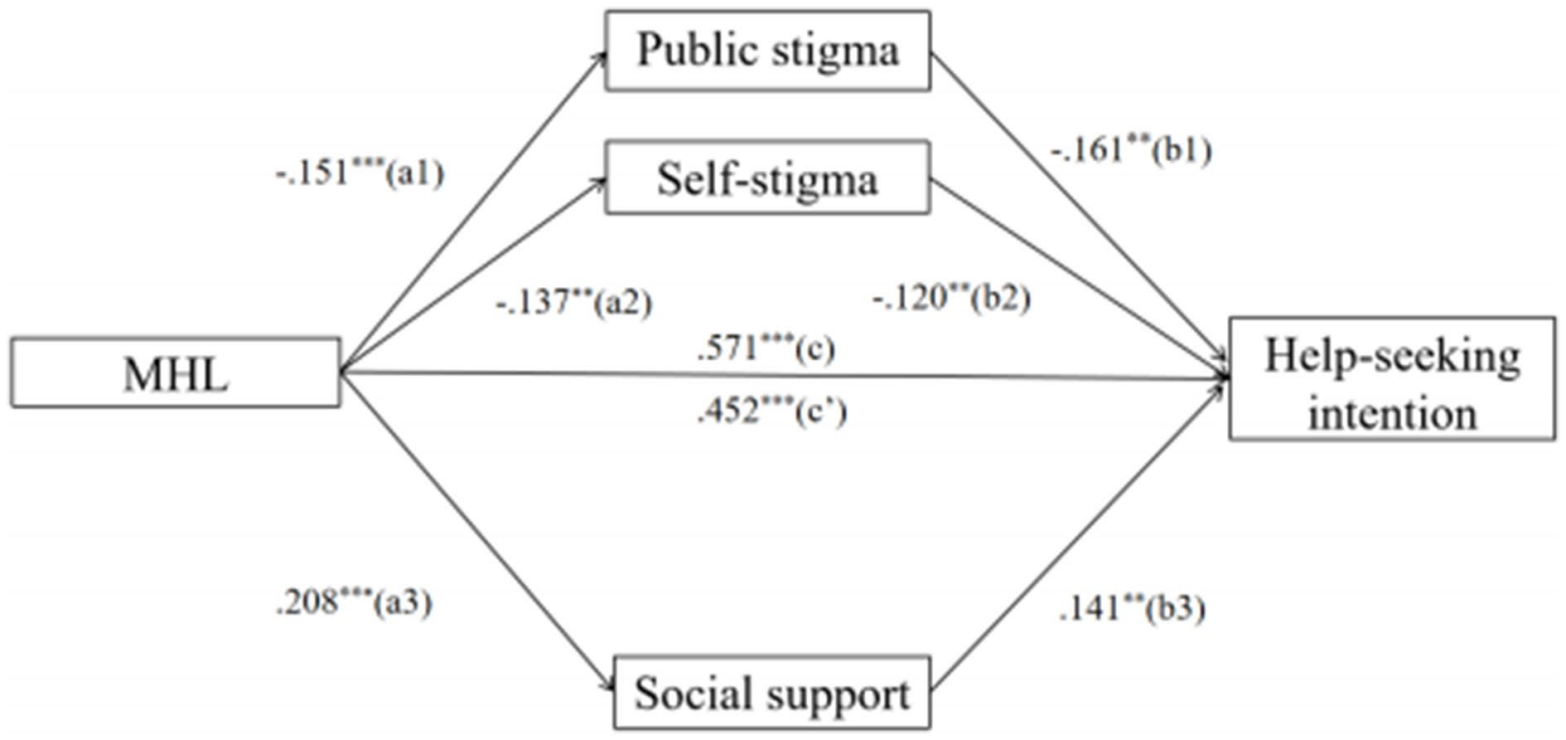
The mediating role of public stigma, self-stigma, and social support in the relationship between MHL and the help-seeking intention in Chinese elite athletes. MHL, mental health literacy; HSI, help-seeking intention; Gender, age, sports type, training years, training level, days of training per week and hours of were controlled in the mediation models.

To test the indirect effects, public stigma, self-stigma, and social support were used as mediating variables, MHL as the independent variable, and HSI as the dependent variable. The indirect effects were shown as follows: (1) the indirect effect of MHL → public stigma → HSI was 0.024 and a 95%*CI* [0.003, 0.053], which accounted for 7.12% of the total effect, the *R_med_^2^* of MHL → public stigma → HSI is 0.018. Specifically, MHL predicted public stigma (Estimate = −0.151, 95%*CI* [−0.187, −0.045], *p* < 0.001), and public stigma predicted HSI (Estimate = −0.161, 95%*CI* [− 0.549, −0.164], *p* < 0.001); (2) the indirect effect of MHL → self-stigma → HSI was 0.016 and 95%*CI* [0.002, 0.038], which accounted for 4.75% of the total effect, the *R_med_^2^* of MHL → self-stigma → HSI is 0.015. Specifically, MHL predicted self-stigma (Estimate = −0.137, 95%*CI* [−0.069, −0.013], *p* < 0.01), and self-stigma predicted HSI (Estimate = −0.120, 95%*CI* [−1.181, −0.186], *p* < 0.01); as well as (3) the indirect effect of MHL → social support → HSI was 0.029 and 95%*CI* [0.009, 0.055], which accounted for 8.61% of the total effect, the *R_med_^2^* of MHL → social support → HSI is 0.027. Specifically, MHL predicted social support (Estimate = 0.208, 95%*CI* [0.018, 0.047], *p* < 0.001), and social support predicted HSI (Estimate = 0.141, 95%*CI* [0.578, 2.442], *p* < 0.01). Additionally, the direct effects from MHL to HSI is β = 0.267, 95%*CI* [0.179, 0.354], *p* < 0.001 (Figure 2).

## Discussion

This study aims to examine the relationship between MHL and HSI, and explored the mediating effects of public stigma, self-stigma, and social support on the relationship between MHL and HSI among Chinese elite athletes. First, the demographic variables of the participants revealed that their average MHL scores were low to moderate. Previous research (Kim et al., 2020) as demonstrated that 92% of Chinese elite athletes had a low-to-moderate level of MHLS score, which is consistent with our study (Han et al., 2019). Similarly, previous research has confirmed that Irish athletes have lower MHL levels with a mean score of 3.7 found by Breslin et al. (2018) than the general population with a mean score of 3.7 revealed by Rüsch et al. (2011). Additionally, the levels of stigma, public stigma, and self-stigma of the participants were moderate-level high, which is consistent with previous research (Han et al., 2019). Indeed, previous studies have demonstrated that the stigmatisation of elite athletes mainly stems from coaches, teammates, family members, and even competitors (Bauman, 2016; Gulliver et al., 2010). These different forms of stigma can hinder elite athletes from seeking help when they suffer from mental health issues. Our study thus further demonstrates the need for actions to reduce different forms of stigma in sports settings. Moreover, the participants had a low to moderate level of HSI, which is consistent with previous research (Han et al., 2019). This finding may be related to athletes’ lower levels of MHL and higher levels of stigma (Gulliver et al., 2012b; Han et al., 2019). In summary, the participants had low levels of MHL and HSI and high levels of public stigma and self-stigma.

Hypothesis 1 was supported. In line with previous research, we found that MHL was significantly positively correlated with HSI (O’Connor and Casey, 2015; Wilson et al., 2005; Smith and Shochet, 2011). Considering the severity of mental health issues and the unlikelihood of seeking help, MHL is crucial for elite athletes. This can not only improve their relevant knowledge but also promote relevant help-seeking attitudes, intentions, and behaviours when athletes experience mental health issues (Gulliver et al., 2012b). This further illustrates the urgent need for relevant MHL interventions in the Chinese elite athlete population (Bu, 2021). Similarly, initiatives to understand the impact of MHL on the need for mental health care have been suggested in sports and other fields. For instance, several studies on MHL have been conducted with coaches, sports staff, teachers, and students, among others (Breslin et al., 2022; Bu et al., 2020; Kutcher et al., 2015, 2016b). Given the importance of promoting MHL development in athletes, emphasis should be placed on creating and implementing evidence-based MHL interventions in the athlete population (Bu et al., 2020, 2023).

Hypothesis 2 was supported. Our results showed that public stigma was significantly negatively linked with the mediating effect of MHL on HSI among elite athletes. It has been suggested that athletes who seek mental health treatment may be recoginsed as weak by others (Bauman, 2016; Gulliver et al., 2010). Meanwhile, MHL was significantly negatively correlated with public stigma. This further supports the findings of previous studies (Han et al., 2019). Therefore, public stigma could be further reduced by improving the level of MHL among athletes. Additionally, due to the high level of public stigma perceived by athletes, there is an urgent need to educate those involved in the sports environment, such as team leaders, coaches, support staff, and parents of athletes (Breslin et al., 2017; Bu et al., 2023; Hurley et al., 2018; Sebbens et al., 2016). Furthermore, public stigma was significantly negatively linked with HSI, which is also consistent with the results of previous studies (Gulliver et al., 2012b; Han et al., 2019). To reduce severe public stigma, some coaches have suggested that mental health should be discussed openly among athletes (Mazzer and Rickwood, 2015). This could help athletes recognise the importance of mental health issues and reduce the level of public stigma at the same time (Mazzer and Rickwood, 2015). In summary, future intervention studies could consider incorporating efforts to reduce public stigma into mental health knowledge, awareness, or literacy sessions. This approach may contribute to fostering athletes’ intention to seek help.

Hypothesis 3 was supported. Specifically, self-stigma was revealed to be significantly negatively linked with the mediating role of MHL on the HSI of elite athletes. As discussed, self-stigma refers to a person’s diminished self-esteem and sense of self-efficacy due to public stigma (Corrigan, 2004). Previous studies have shown that compared to non-athletes, athletes experience higher degrees of self-stigma (Watson, 2005). As discussed, these higher degrees of self-stigma may affect the decision-making and service selection stages by either facilitating or discouraging these processes (Cauce et al., 2002). Additionally, it has been shown that the male athlete population has higher levels of self-stigma, which may be related to their masculinity (Levant et al., 2013; Seidler et al., 2016). Meanwhile, our study showed that the MHL level of elite athletes was significantly negatively correlated with the level of self-stigma, which further supports the findings of previous studies (Han et al., 2019). Due to self-stigma, athletes are less likely to report their awareness of mental health issues because doing so can make them feel ashamed, embarrassed, or uncomfortable (Bu, 2021). Therefore, it is also necessary for athletes to improve their MHL and education related to mental health to reduce their level of self-stigma. Furthermore, similar to previous research, our study showed that the level of self-stigma among elite athletes was significantly negatively linked with HSI (Han et al., 2019). As stigma was more severe in the athlete population, this also discouraged seeking help. For example, in a group of male professional soccer players, those with high-risk mental health issues did not seek professional support, citing the main barriers as stemming from their self-stigma and the belief that seeking help was a choice only made by those who were desperate (Wood et al., 2017). Additionally, one study (Swann et al., 2018) found that the self-stigma of masculinity reduced the likelihood of seeking help among adolescent male athletes, and Hinton et al. (2006) found that men were less likely than women to pay attention to their mental health issues, such as depression, and less proactive than women in dealing with depression. This tendency was connected with their higher degrees of self-stigma. Therefore, future research should urgently prioritise interventions to reduce the levels of self-stigma among athletes. For example, tailored MHL interventions could be incorporated to reduce self-stigma, thereby promoting HSI and help-seeing behaviour (Bu et al., 2023). More attention should also be given to addressing self-stigma among male athletes (Wood et al., 2017).

Hypothesis 4 was also supported. Our study demonstrated that the mediating effect of social support on the relationship between MHL and athletes’ HSI was positive and significant. This demonstrated that MHL was significantly and positively linked with social support, while social support was significantly and positively linked with HSI, which is consistent with previous research (Rafal et al., 2018). For example, coaches are among the most significant providers of social support for athletes. When coaches believe that athletes need to emphasise and address the mental health issues they may be experiencing, athletes are more prone to seek assistance (Gulliver et al., 2012b). Another helpful source of support for athletes is parental support. When athletes experience mental health issues, adequate parental support is crucial for athletes (Mason et al., 2015). Parental support produces a range of positive outcomes for athletes, including creating a loving environment and providing helpful advice (Dorsch et al., 2017; Swann et al., 2018). Furthermore, for athletes, building strong, positive relationships with teammates can provide a platform for discussing mental health issues when they arise. For example, team activities coupled with supportive attitudes and behaviours were identified by some athletes as beneficial. Emotional support among teammates played a facilitating role in this regard. Consequently, the importance of teammates and the support they provide emerges as one of the main sources of social support (Rickwood et al., 2005; Swann et al., 2018). Furthermore, from the perspectives of both coaches and athletes, appropriate professional support from sports psychologists and clinical psychologists is an effective means of assisting athletes. Support from sports psychologists, in particular, may also encourage athletes to seek help (Biggin et al., 2017). For example, when athletes do not have a well-established relationship with their sports psychologists, they are less likely to approach them when they need assistance with mental health issues, and vice versa (Biggin et al., 2017). Social support can thus facilitate help-seeking among elite athletes, and different types of support resources are highly suggested.

From the practical perspective, the enhancements of public-and self-stigmas, as well as social support are significant in the MHL interventions. All of the MHL components, including risk factors, attitudes toward mental health issues, and the identification of specific symptoms, were included in the intervention’s content (Gorczynski et al., 2019; Gulliver et al., 2012b; Spiker and Hammer, 2018). Additionally, the content of stigma and its detrimental impact on mental health and help-seeking, as well as the importance of social support, also need to be included in mental health literacy interventions (Breslin et al., 2022; Bu et al., 2020; Kutcher et al., 2016a). Reduced both public-and self-stigmas, and increased social support may influence future behavioural responses.

The strengths of this study include the following. First, to the best of our knowledge, this study is the first to examine the relationships between public stigma, self-stigma, and social support through which MHL affects HSI among Chinese elite athletes. The relationships between these variables have not been previously tested, as no previous studies have been conducted on mediation in the elite athlete population. In light of early intervention and the diagnosis of mental health issues, the association between MHL, stigmas, social support, and HSI is significant. It also provided the empirical support for future mental health policy recommendations and help-seeking campaigns. Second, the participants involved in this study were all Chinese elite athletes, of which 63.78% competed at the international level. This can better reflect the meditating effects through which MHL affects HSI among Chinese elite athletes. Third, there were no missing data in this study as all data were collected through a website, offering a convenient and efficient research method.

This study has several limitations. First, we used a cross-sectional design and self-reported questionnaires to test the relationships among MHL, public-and self-stigmas, social support and HSI. It allows for a more cautious interpretation of causal relationships between key variables. Another key issue related to the cross-sectional design is that the mediation analysis may be biased and the mediating variables may not have mediating effects in longitudinal analyses (Maxwell et al., 2011). Future studies should consider alternative mediation models, such as variations of autoregressive models or the use of longitudinal studies, to better explore the relationships between variables. And the self-reported questionnaires may lead to methodological bias, and response bias (Podsakoff et al., 2003). To avoid such bias, future studies should consider preventive methods, such as using MHL as a predictor and mediator; applying other remedial measures, such as maximising respondent motivation and minimising task deficits; and considering some physiological indicators to assess the results (Podsakoff et al., 2003; Taris and Kompier, 2014). And social desirability bias also remains (Podsakoff et al., 2003). The tendency to overstate more desirable characteristics and neglect to report socially unfavourable attitudes and actions is known as the social desirability bias (McCaffery, 2018). Paulhus (1984) theory of social desirability bias proposes two elements. One is impression management, which is the deliberate presentation of oneself to others or to fit into a specific situation. The second component is self-deception, which is motivated by the desire to uphold a favourable self-concept and may be unconscious. Based on a thorough analysis of the literature, Tourangeau and Yan (2007) demonstrated that the need to manage impressions and prevent shame and consequences from revealing sensitive information frequently drives socially acceptable response bias. Therefore, it might impact the results of self-reported measures of stigma and social support. Future research should therefore include manipulation checks to see whether participants comprehend the survey’s content to prevent social desirability bias. Second, the 450 participants recruited in the study, the age range of them (16 to 29 years) is quite large, and the number of athletes from individual events in this study was much higher than the number of athletes from group events, and the proportion of male athletes was higher than that of female athletes. This was due to convenience sampling. The main aim is to examine the relationships among MHL, stigmas, social support, and HSI in elite athletes, therefore, we did not further test the differences between adolescence and adulthood athletes’ groups. The developmental differences between these groups might exist. Future studies might consider age, to test the relationships among those variables. And as athletes from group events also have mental health issues, future studies should recruit more athletes from group events to the mediating effects. Although female athletes have been shown to have higher levels of MHL and HSI than male athletes (Han et al., 2019), lower levels of MHL in female athletes also exist. Therefore, future studies should include more female athletes. Third, the assessment of the social support variable in this study was adapted from existing research. Future studies should use questionnaires that have been tested for psychometrics, thus improving the reliability and validity of the scale. And this study only assessed HSI and did not assess help-seeking behaviours. Help-seeking theory (Rickwood et al., 2005) illustrates the close relationship between intention and behaviour, thus this study focused on assessing HSI. However, based on the Theory of Planned Behaviour (Ajzen, 1991), some studies have suggested that other factors between intention and behaviours need further exploration. Future research should thus assess help-seeking behaviour in athletes. Fourth, although the hypotheses were all supported, it should be noted that the effect size of paths from MHL → public stigma → HSI, MHL → self-stigma → HSI, and MHL → social support → HSI were small. This may be due to other possible reasons, such as prior help-seeking experience and a tendency to self-help (Osman et al., 2023). Since this study included only some of the above mediating variables, which do not represent a comprehensive assessment of such variables. Additional mediating variables, such as prior help-seeking experience, a tendency to self-help, help-seeking attitudes, self-efficacy, excellence, resilience, and the fear of failure should be included to test the mediating effects of different variables in future research. Additionally, a randomised controlled trial design is recommended for future research to test the causal effect explaining the mediating effects of MHL on help-seeking in a sports environment. Fifty, disabled elite athletes were not included in our study, which may limit the generalization of study. Since disabled athletes suffer from mental health issues even more than non-disabled (Reardon et al., 2019; Watson and Vehmas, 2014), it should be paid more attention in those individuals. Sixth, Spiker and Hammer (2018) have suggested that MHL can be a theory to guide future studies to explain the important constructs and their interrelationships. However, our study only focused on the narrow definition of MHL. Since the definition of MHL has been evolving, future studies might focus on other aspects, such as negative and positive mental health knowledge. Furthermore, due to some differences between the Chinese version of MHLS with 33-item and the English version of MHLS with 35-item (O’Connor and Casey, 2015), it is impossible to compare the level of MHL among Chinese elite athletes and in the United States. Future studies might consider using other scales to evaluate MHL in Chinese elite athletes.

## Conclusion

This study examined the relationship between MHL and HSI, and explored the mediating effects of public stigma, self-stigma, and social support on the relationship between MHL and HSI among Chinese elite athletes. The results showed a significant positive correlation between MHL and HSI. Furthermore, public stigma, self-stigma, and social support emerged as significant mediators, shedding light on how MHL affects HSI. These findings provide empirical support for the mechanisms of behavioural change through which MHL leads to HSI. This can assist future MHL interventions for elite athletes.

## Data Availability

The raw data supporting the conclusions of this article will be made available by the authors, without undue reservation.
